# Spatial distribution of mortality from colorectal cancer in the southern region of Brazil

**DOI:** 10.1371/journal.pone.0288241

**Published:** 2023-07-07

**Authors:** Matheus Jacometo Coelho de Castilho, Miyoko Massago, Carlos Eduardo Arruda, Matheus Henrique Arruda Beltrame, Eleanor Strand, Carlos Edmundo Rodrigues Fontes, Oscar Kenji Nihei, Rogério do Lago Franco, Catherine Ann Staton, Raissa Bocchi Pedroso, Luciano de Andrade

**Affiliations:** 1 Postgraduate Program in Health Sciences, State University of Maringa, Maringa, Parana, Brazil; 2 Postgraduate Program in Management, Technology and Innovation in Urgency and Emergency, State University of Maringa, Maringa, Parana, Brazil; 3 Department of Medicine at the State University of Maringa, Maringa, Parana, Brazil; 4 Department of Emergency Medicine, Duke University School of Medicine, Durham, North Carolina, United States of America; 5 Center of Education, Literature and Health, Western Paraná State University, Foz do Iguaçu, Parana, Brazil; PLOS ONE, UNITED KINGDOM

## Abstract

Colorectal cancer (CRC) is the leading cause of death due to cancer worldwide. In Brazil, it is the second most frequent cancer in men and women, with a mortality reaching 9.4% of those diagnosed. The aim of this study was to analyze the spatial heterogeneity of CRC deaths among municipalities in south Brazil, from 2015 to 2019, in different age groups (50–59 years, 60–69 years, 70–79 years, and 80 years old or more) and identify the associated variables. Global Spatial Autocorrelation (Moran’s I) and Local Spatial Autocorrelation (LISA) analyses were used to evaluate the spatial correlation between municipalities and CRC mortality. Ordinary Least Squares (OLS) and Geographically Weighted Regression (GWR) were applied to evaluate global and local correlations between CRC deaths, sociodemographic, and coverage of health care services. For all age groups, our results found areas with high CRC rates surrounded by areas with similarly high rates mainly in the Rio Grande do Sul state. Even as factors associated with CRC mortality varied according to age group, our results suggested that improved access to specialized health centers, the presence of family health strategy teams, and higher rates of colonoscopies are protective factors against colorectal cancer mortality in southern Brazil.

## Introduction

Accounting for around 10 million deaths (nearly one in six deaths) in 2020, cancer still remains the second leading cause of death worldwide [1]. Among 1.93 million people diagnosed with cancer colorectal (CRC), over 900,000 died due to this disease in 2020 [1]. This corresponds to almost 10% of total cancer deaths. In addition, an estimated two million people are diagnosed with CRC each year [2, 3]. According to Center of Diseases Control and Prevention (CDC), the risk factors of CRC are aging; inflammatory bowel diseases; occurrence of CRC in the family and genetics; physical inactivity; low ingestion of fruits; vegetables, and fiber; obesity, and consumption of licit drugs, such as tobacco and alcohol [4].

Early diagnosis by techniques, such as screening by colonoscopy and blood exam, associated with the treatment like surgery, radiotherapy, and drug therapy can reduce up to 68% of CRC deaths worldwide [1, 5–7]. However, in developing countries, healthcare services are not equitably distributed; thus, regions with limited or no access to oncology services experience higher cancer mortality rates [8].

In most high-income countries (HIC), oncology services are available and accessible and lead to a reduction of CRC deaths, alongside improvements in cancer prevention and treatment [9]. In contrast, limited healthcare services, and lack of resources dedicated to the aging population [10–13], have increased CRC mortality rates in most low- and middle-income countries (LIMC) [14].

In Brazil, an LMIC in South America, 450,000 new cancer diagnoses are projected between 2020 and 2022, 41,000 of which would be CRC [15]. In general, the South region of Brazil, despite its high Human Development Index (HDI) (indicates high life expectancy at birth, literacy, and income), has the highest cancer incidence (23.4%), predominantly in the prostate, breast, lung, and intestinal [15, 16]. The CRC incidence in South Brazil raised from 22.8 cases per 100,000 inhabitants in 2005 to 113.2 cases per 100,000 inhabitants in 2018, although the lethality decreased from 12.2 cases per 100,000 inhabitants in 2005 to 5.5 cases per 100,000 inhabitants in 2018 [17], indicating that the treatment of this type of cancer is effective.

In addition to an inequitable distribution of cancer burden [18–20], inequities in the distribution of healthcare services, professional qualifications, time between diagnosis and treatment, and underreporting to the health information system can also influence the CRC mortality rates across Brazil [20–24]. As a continental country, some differences of HDI and consequently, access to healthcare services can be noticed across Brazilian municipalities of the same geographical region, but usually, in the Northeast region of the country, municipalities tend to have a low HDI and limited health services, with little or none specialized care. Municipalities in the Midwest, Southeast, and North regions have a higher HDI, but health services are still limited. The South region´s municipalities have a high HDI and adequate health services, including specialized care, a factor that can contribute to low mortality due CRC. However, individual factors such as habits, health behaviors, and cultural issues, like resistance to seeking preventive care, remain barriers to adequate care [16, 17, 21–25]. There is a lack of studies in the South of Brazil regarding geographical accessibility, barriers, and spatial heterogeneity to healthcare and CRC early diagnosis and treatment, and how this influences mortality at the municipal level.

Spatial analysis has also been used to study the CRC profile in other countries [26–29]. However, as far as we know, this study is a pioneer in two aspects: 1) analyzing the heterogeneity of spatial distribution of colorectal cancer mortality in Brazil, and 2) describing its associations with socioeconomic factors and accessibility to health care services.

## Methods

### Study design and location

This is an ecological, observational, cross-sectional study following the Strengthening the Reporting of Observational Studies in Epidemiology (STROBE) guidelines. STROBE guidelines ensure transparent reporting and are considered the standard for observational studies [30].

The Southern region of Brazil comprises the states of Paraná, Santa Catarina, and Rio Grande do Sul. It is 576,736,815 km^2^, the smallest region in terms of total area, and has a total of 27,386,891 inhabitants, distributed across 1,191 municipalities with an average HDI of 0.754 in 2010, the third highest of the Brazilian regions [31].

**Fig 1:**
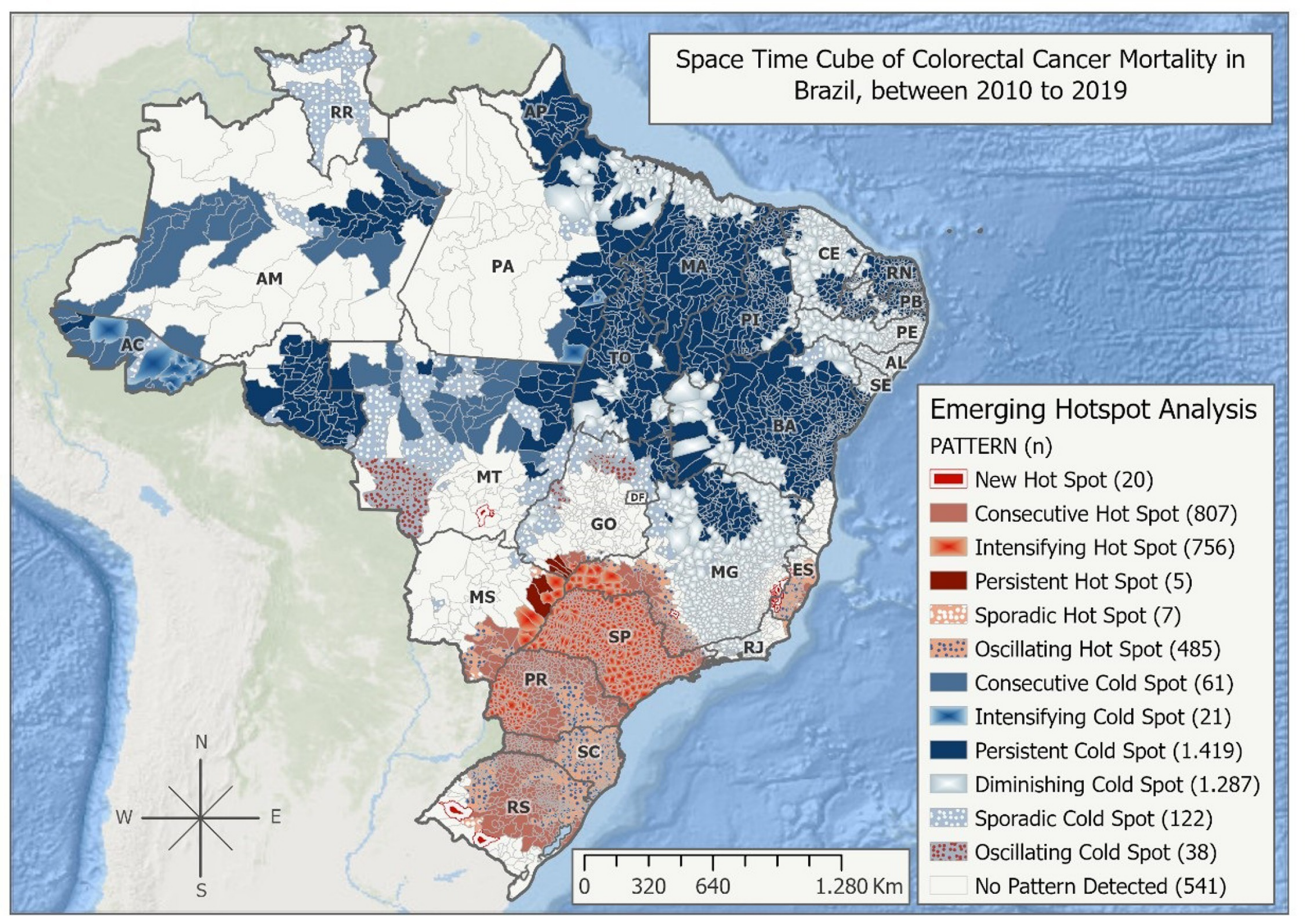
Emerging hotspot analysis pattern of colorectal cancer mortality in Brazil, between 2010 and 2019.

This region was selected, because in a decade time evolution (2010 to 2019), the Southern region experienced significant increases in colorectal cancer mortality (trend statistic = 3.398; p-value = 0.0007), as illustrated in the Space-Time Cube [32, 33] (Fig 1).

### Data and variables sources

CRC mortality data were available through the Mortality Information System of the Information Technology Department of the Unified Health System (DATASUS), available from https://datasus.saude.gov.br/mortalidade-desde-1996-pela-cid-10. CRC deaths were defined and extracted according to the International Statistical Classification of Diseases and Related Health Problems (ICD-10). ICD-10 codes included malignant neoplasm of the colon (C18), malignant neoplasm of the rectosigmoid junction (C19), and malignant neoplasm of the rectum (C20) [31, 34]. These data were obtained for four age groups (50 to 59, 60 to 69, 70 to 79, and 80+) between 2015 and 2019. Income, educational level, and coverage of the Family Health Strategy team (FHS) (primary healthcare coverage) secondary data were obtained from the Brazilian Institute of Geography and Statistics (IBGE) [31]. Population data in South Brazil between 2010 and 2019, according to age groups (50 to 59, 60 to 69, 70 to 79, and 80+) by municipality, was obtained from IBGE [31].

The absolute number of procedures, used to calculate rates of surgeries and colonoscopies, was obtained from DATASUS data linked to the Hospital Admissions System (SIH). This was done through the “Procedures Performed” field of the Hospital Admission Authorizations (AIH) and was filtered by the standardized codes of the Brazilian Unified Health System [35], as has been described in Box 1. All data used in the study are free and publicly available through DATASUS (https://datasus.saude.gov.br/informacoes-de-saude-tabnet/) and IBGE (https://www.ibge.gov.br/cidades-e-estados.html?view=municipio).

**Box 1. Codes of procedures, medicines, and office of personnel management system (SIGTAP) of colorectal procedures, according to Brazilian Unified Health System.**

**Table:** 

**Procedure code**	**Procedure**
02.09.01.002–9	Colonoscopy
04.07.02.006–3	Partial colectomy (hemicolectomy)
04.07.02.007–1	Total colectomy
04.07.02.008–0	Video laparoscopic colectomy
04.07.02.040–3	Abdominal Rectosigmoidectomy
04.07.02.041–1	Abdominoperineal rectosigmoidectomy
04.16.05.001–8	Abdominoperineal amputation in oncology
04.16.05.002–6	Partial colectomy (hemicolectomy) in oncology
04.16.05.003–4	Total colectomy in oncology
04.16.05.005–0	Local excision of rectal tumor in oncology
04.16.05.007–7	Abdominal rectosigmoidectomy in oncology
04.16.05.006–9	Total proctocolectomy in oncology (revoked in 2013)
04.16.05.011–5	Total proctocolectomy in oncology

Health care accessibility was estimated in each municipality by colorectal procedure rates (colonoscopies and surgeries) and percentage area covered by FHS (primary healthcare coverage) between 2010 and 2019. Socioeconomic indicators were per capita income and education level up to 8^th^ grade according to the last census (2010) [31].

### Data analysis

#### Spatial distribution

The mortality rate calculated according to age group, multiplied by 100,000 age-adjusted inhabitants, was smoothed using the Empirical Bayesian Estimator based on the "Queen" type neighborhood matrix, for each municipality in the state, through the Geoda™ software, version 1.12.0 [36].

To evaluate the spatial correlation, first, we used the Moran’s Index (Moran’s I). This analysis measures the overall spatial autocorrelation of the dependent variable in areas from specific geographic regions (i.e., municipalities). Given a set of features and an associated variable, the Moran’s I assess whether the pattern is positively (Moran’s I> 0) or negatively (Moran’s I <0) clustered, or is randomly scattered (Moran’s I = 0) [37].

One limitation of Moran’s Index is that it can hide local spatial association patterns since values close to zero of Moran’s I do not always indicate the absence of spatial correlation at the local level [38]. To overcome this limitation, a local spatial association indicators (LISA) analysis was performed in each municipality to find statistically significant spatial clusters (95% confidence level; p < 0.05) [39].

LISA analysis allows us to see if regions with high rates of a specific event, such as CRC deaths, were surrounded by municipalities with same patterns, namely hotspots or clusters of high mortality rates (high-high clusters), or if regions with low CRC deaths rates were surrounded by municipalities with same patterns, namely cold spots or clusters of low lethality rates (low-low clusters) [25].

For better visualizations, we plot choropleth maps of smoothed rate (CRC mortality rate by 100,000 inhabitants) and local spatial association indicators (LISA) to investigate the occurrence of clusters [39].

#### Spatial regression

We selected a group of variables with multicollinearity condition numbers lower than 30 [40]. This way, for each age group, the following variables remained for the Ordinary Least Squares Regression (OLS) and geographically weighted regression (GWR) models: accessibility to oncology center, per capita income, coverage of Family Health Strategy team, educational level, and colonoscopy and surgery rate [41].

The Ordinary Least Squares Regression (OLS), performed by GeoDa software v. 1.12.0 [42, 43], and Geographically Weighted Regression (GWR), performed by software GWR program, version 4.0 [44, 45], were used to explore the relationships between the dependent and independent variables. The choropleth maps were generated in the software QGIS software version 2.14 [46].

OLS analysis attempts to understand the global relationship between dependent and independent variables, assuming that associations between the variables can be heterogeneous across the studied area (i.e., can change according to geographical region) (39). For this analysis, we considered statistically different t-values lower than -1.96 (t< -1.96) or higher than +1.96 (t>+1.96) [47].

Geographically weighted regression (GWR) belongs to a group of local modeling techniques that fit a regression model to each geographic location based on neighbors within a specific area and use the distance in a weight-dependent function [48]. This approach has the advantage of avoiding abrupt changes in the local statistics calculated for adjacent areas, helps visualize the spatial variability within the geographic area, and allows for analysis of regionally aggregated data [47].

GWR analysis produces an estimate for the association between CRC mortality and its variables for each municipality. Coefficients of each variable that were significant in the global model were therefore used to determine the impact of space on the results [49]. The performance of the GWR model was evaluated based on the adjusted R2 indicators and residual Moran’s I parameters of both models for general adjustment. The lower the value of these metrics, the higher the correlation. In addition, the Akaike Information Criterion (AIC) was considered to evaluate the quality of each statistical model as well as to select the model.

### Ethical aspects

In accordance with Resolution No. 510/16 of the National Health Council, an exemption was granted by the Standing Committee on Ethics in Research Involving Human Beings of the State University of Maringá, considering we used secondary, publicly available data sources.

## Results

Between 2015 and 2019, 18,956 deaths due to CRC were recorded. Of these, 16,981 occurred in people aged 50+, with an average age of 71.24± 11.01 years. Rio Grande do Sul recorded 8,552 deaths (50.36%), Paraná 5,445 deaths (32.06%) and Santa Catarina, 2,984 (17.57%). CRC deaths per age group 50 to 59, 60 to 69, 70 to 79, and 80+ were 2,892 (17.03%), 4,682 (27.57%), 5,069 (29.79%), and 4,338 (25.54%), respectively.

**Fig 2:**
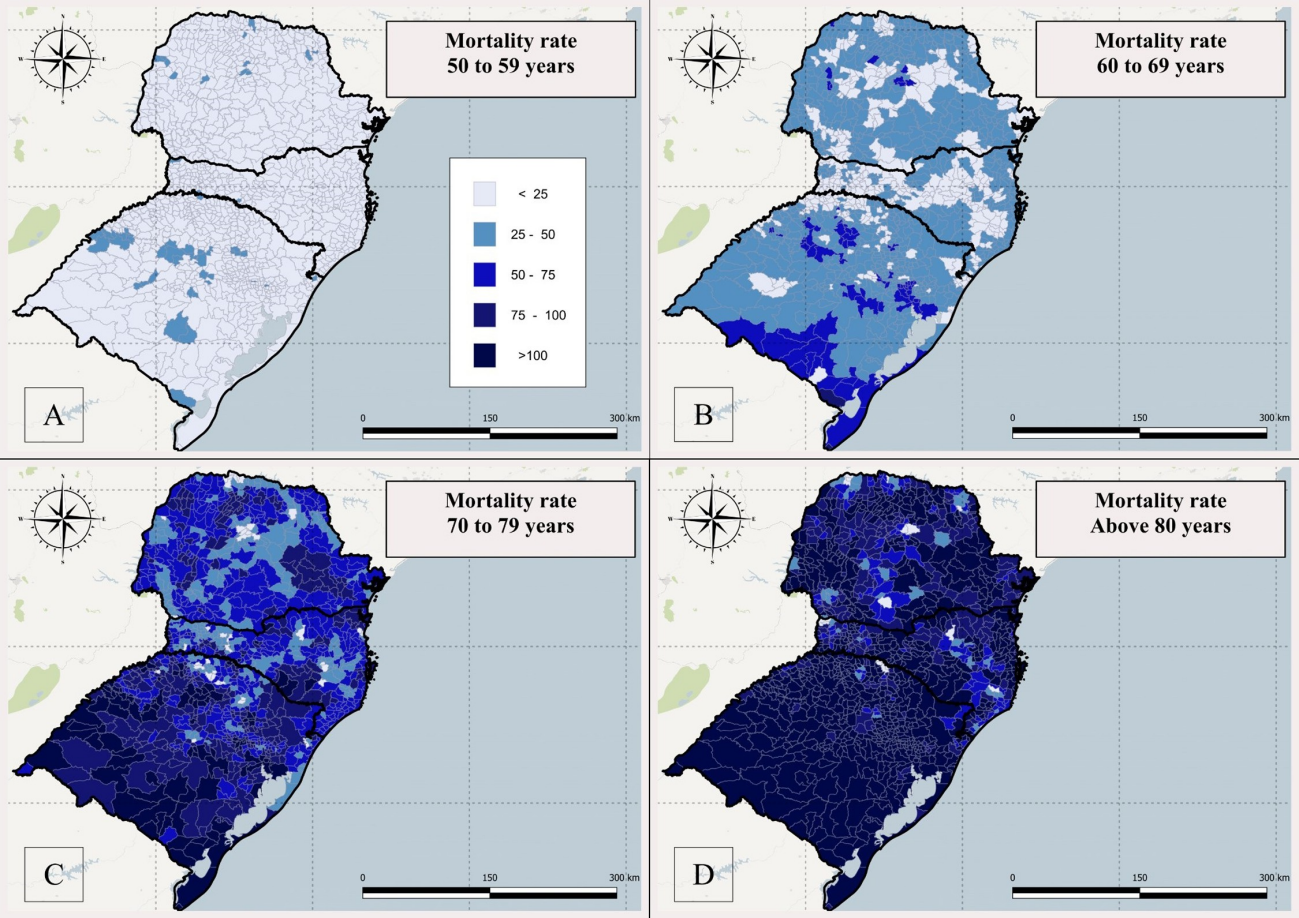
Spatial distribution of the smoothed rate of colorectal cancer deaths per 100,000 inhabitants in southern Brazil, from 2015 to 2019.

For the 50 to 59 age group, CRC mortality rate per 100,00 inhabitants ranged from 15.4 to 35.0, with 95.21% (1134/1191) municipalities with zero to 24.9 deaths per 100,000 inhabitants (Fig 2A). For the 60 to 69 age group, this rate changed from 35.4 to 87.8, with 61.96% (738/1191) municipalities with 25.0 to 49.9 deaths per 100,000 inhabitants (Fig 2B). For the 70 to 79 age group, this rate changed from 60.0 to 179.0, with 68.09% (811) municipalities with 50.0 to 99.9 deaths per 100,000 inhabitants (Fig 2C). For the 80+ age group, this rate changed from 129.0 to 299.0, with 65.99% (786/1191) of municipalities showing mortality over 100 deaths per 100,000 inhabitants with 80+ (Fig 2D).

The Moran’s Index showed a positive spatial autocorrelation in all four age groups (p<0.001). Correlations were 0.589, 0.646, 0.663 and 0.667, for the 50 to 59, 60 to 69, 70 to 79, and 80+ age groups, respectively. This indicates that municipalities with high CRC mortality rates tend to be surrounded by other municipalities with similar characteristics.

**Fig 3:**
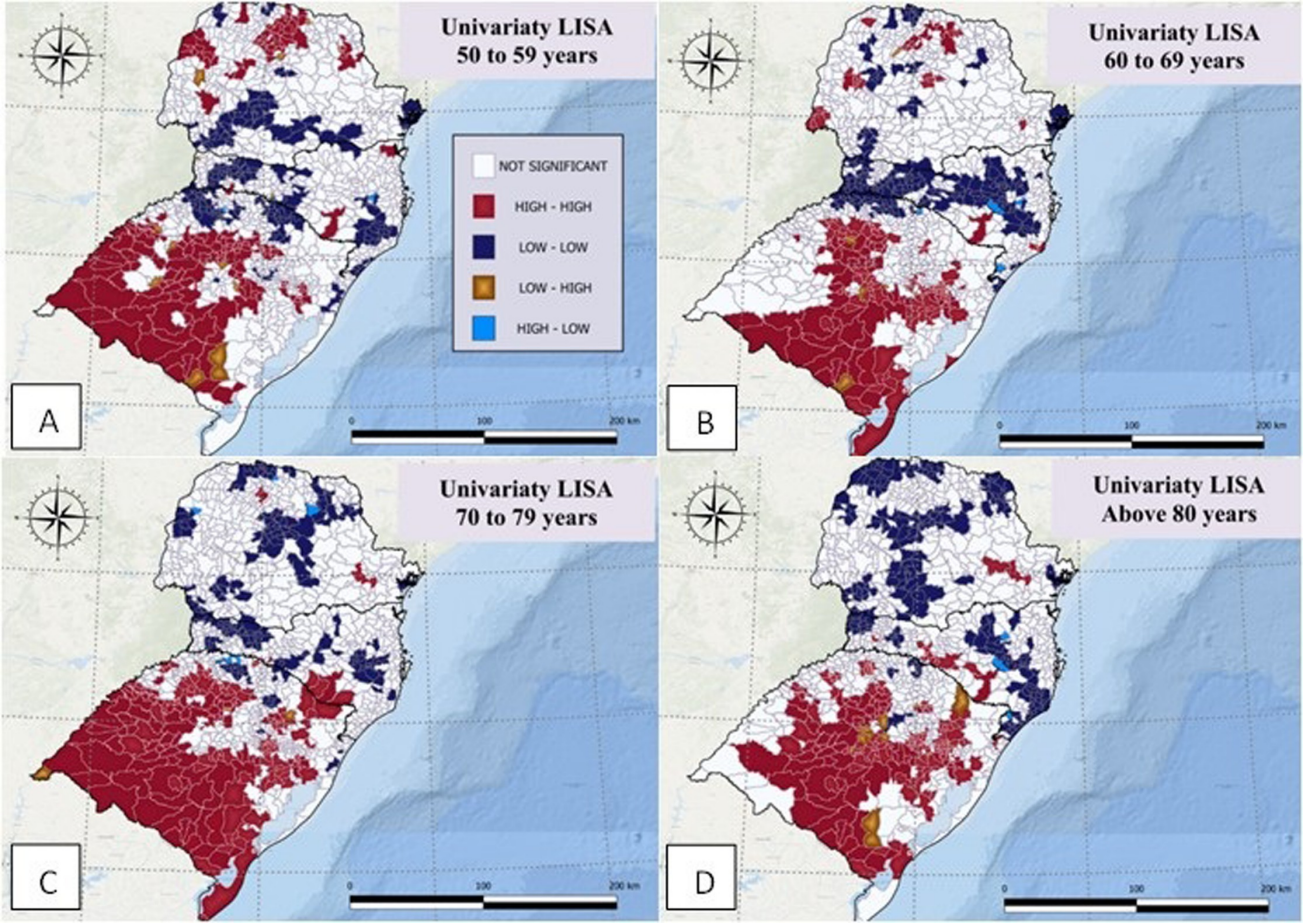
Local spatial association indicators (LISA) to identify possible deaths by colorectal cancer clusters, in Southern Brazil, between 2015 and 2019, for people aged 50 to 59 years (A), 60 to 69 years (B), 70 to 79 years (C), and 80 years old or above (D).

High-high clusters were found in 15.3% (182/1191) municipalities for the 50 to 59 years age group. These clusters were located in the North, Northwest, Northeast, and Southwest of Paraná; Southeast of Santa Catarina; and Central, South, West, and Southeast of Rio Grande do Sul (Fig 3A).

In the 60 to 69 years age group, high-high clusters occurred in 15.0% (179/1191) of municipalities. These clusters were located in the central, north, northwest, west and a small part of east of Paraná, south and southeast of Santa Catarina, and northwest, northeast, center, south, and southeast of Rio Grande do Sul (Fig 3B).

For the 70 to 79 years age group, 14.5% (173/1191) of municipalities showed high-high clusters. These clusters were located in a small part of north and southeast of Paraná, southeast of Santa Catarina, and northwest, northeast, west, center, all regions of southwest to southeast of Rio Grande do Sul (Fig 3C). In the 80+ years age group, high-high clusters occurred in 15.1% (180/1191) of municipalities located in a small part of southeast of Paraná, a small part of south to southeast of Santa Catarina, and a small part of north and northeast, almost all central region, and south to southeast of Rio Grande do Sul (Fig 3D). In dimension, the 70 to 79 years old showed the highest area of high-high clusters and 60 to 69 years old the least.

Regarding our regression analysis, the result of 21.6 (conditional number test–GeoDa) indicates low multicollinearity of the tested variables; thus, it is not influencing the regression results. Negative correlations (protective effects) between CRC mortality and FHS was found in the 50 to 59 (t = -3.71), 60 and 69 (t = -5.57), 70 to 79 (t = -5.87), and 80+ (t = -5.77) years age groups. Colonoscopy rate was negatively correlated in the 70 to 79 (t = -2.00) and 80+ (t = -2.44) age groups (Table 1).

**Table 1:** Ordinary least-squares (OLS) and geographically weighted regression (GWR) spatial regression results stratified by age-group.

	50 to 59 years									60 to 69 years								
	OLS (Global)				GWR (Local)					OLS (Global)				GWR (Local)				
	Coef.	SD	t	p	Min	1stQu	Median	3rd	Max	Coef.	SD	t	p	Min	1stQu	Median	3rd	Max
**Constant**	13.20	1.22	**10.84**	0.00	-26.29	3.58	10.45	16.50	47.73	30.27	2.40	**12.63**	0.00	-119.86	12.34	23.97	35.35	67.02
**Access.**	373.73	218.68	1.71	0.09	-20,514.2	-2,555.6	1,029.3	4,530.1	30,660.0	1,351.2	430.34	**3.14**	0.00	-24,938.2	-7,560.4	-3,118.0	7,704.1	61,823.4
**Income**	0.00	0.00	-1.32	0.19	-0.02	0.00	0.00	0.01	0.02	0.00	0.00	-1.07	0.28	-0.04	-0.01	0.00	0.01	0.04
**FHS**	-4,356.2	1,175.7	**-3.71**	0.00	-18,472.2	-3,893.8	334.3	3,864.3	18,574.7	-12,834.0	2,303.3	**-5.57**	0.00	-25,521.3	-7,537.5	-539.0	6,429.1	23,562.7
**Schooling**	7.06	2.69	**2.63**	0.01	-32.42	-1.77	6.04	14.10	37.17	14.20	5.28	**2.69**	0.01	-48.85	-0.04	12.37	28.54	98.38
**Surg. Rate**	0.11	0.07	1.59	0.11	-0.73	-0.17	0.02	0.25	1.01	0.28	0.08	**3.44**	0.00	-1.26	-0.04	0.15	0.34	1.19
**Colon. Rate**	0.00	0.00	-0.80	0.43	-0.10	-0.01	0.00	0.02	0.13	-0.01	0.01	-1.54	0.12	-0.16	-0.02	0.00	0.02	0.09
**AIC**	7655.77				7249.269					9262.69				8738.734				
**Adj. R2**	0.024				0.365					0.055				0.445				
**Res. Moran I**	0.556				0.319					0.587				0.321				
	**70 to 79 years**									**≥ 80 years**								
	**OLS (Global)**				**GWR (Local)**					**OLS (Global)**				**GWR (Local)**				
	**Coef.**	**SD**	**t**	**p**	**Min**	**1stQu**	**Median**	**3rd**	**Max**	**Coef.**	**SD**	**t**	**p**	**Min**	**1stQu**	**Median**	**3rd**	**Max**
**Constant**	55.61	4.64	**11.97**	0.00	-138.05	30.14	51.65	72.72	223.80	97.31	8.59	**11.33**	0.00	-133.00	43.06	84.42	110.39	323.46
**Access.**	8,198.1	829.43	**9.88**	0.00	-99,329.8	-13,844.7	-1,061.8	13,844.5	108,643	21,266.8	1,539.5	**13.81**	0.00	-108,663	-23,109.2	3,219.7	28,697.7	152,467
**Income**	0.00	0.00	-0.74	0.46	-0.08	-0.01	0.00	0.02	0.08	0.00	0.01	-0.34	0.74	-0.11	-0.03	-0.01	0.02	0.09
**FHS**	-26,221.0	4,463.7	**-5.87**	0.00	-64,018.7	-22,158.2	-8,394.6	6,095.6	73,800.8	-47,818.3	8,286.4	**-5.77**	0.00	-197,756	-20,018.1	1,669.1	28,699.3	96,229.5
**Schooling**	27.54	10.20	**2.70**	0.01	-121.95	-20.53	22.82	52.97	200.89	34.27	18.96	1.81	0.07	-243.45	3.83	53.20	106.69	292.93
**Surg. Rate**	0.48	0.11	**4.38**	0.00	-1.40	-0.07	0.24	0.55	2.06	0.49	0.14	**3.53**	0.00	-1.80	-0.21	0.12	0.49	1.63
**Colon. Rate**	-0.03	0.01	**-2.00**	0.05	-0.35	-0.05	0.00	0.06	0.23	-0.09	0.04	**-2.44**	0.01	-0.53	-0.15	-0.05	0.06	0.51
**AIC**	10826.7				10378.033					12301.8				11945.674				
**Adj. R²**	0.121				0.458					0.173				0.444				
**Res. Moran I**	0.557				0.292					0.561				0.36				

Coef., coefficient; SD, standard deviation; t, t-value statistics; p, p-value statistics; Constant, dependent variable (CRC mortality); Access., accessibility to treatment centers; FHS, family health strategy coverage; Surg. Rate, surgery rate; Colon. Rate, colonoscopy procedures rate; AIC, Akaike information criterion; Adj. R², Adjusted R-squared; Res. Moran I, residual Moran I.

The associations were positive between CRC mortality and accessibility to oncology center of people aged 60 and 69 (t = 3.14), 70 to 79 (t = 9.88) and 80+ (t = 13.81); schooling for 50 and 59 (t = 2.63), 60 and 69 (t = 2.69), and 70 to 79 (t = 2.70); and surgery rate for 60 to 69 (t = 3.44), 70 to 79 (t = 4.38) and 80+ (t = 3.53) (Table 1).

Correlations between CRC mortality and independent variables were better explained by GWR than OLS for all age groups studied, given the GWR analysis presented higher R^2^, lower AIC, and improvement in residual Moran I (Table 1).

**Fig 4:**
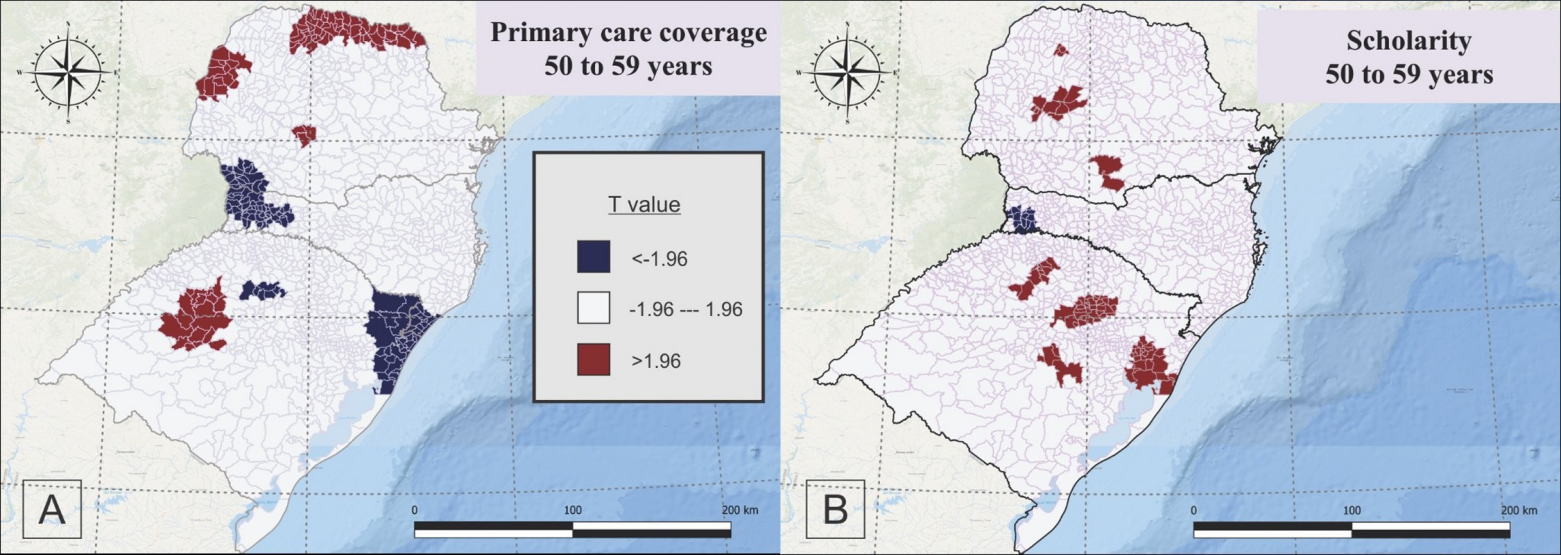
Geographically Weighted Regression for primary care coverage (A) and schooling (B) for people aged 50 to 59 years old, who died due to colorectal cancer, in South Brazil, between 2015 and 2019.

The GWR analysis for people aged 50 to 59 years showed a positive correlation between primary healthcare coverage (FHS) with CRC mortality in north to northeast, west and a few municipalities of center-south of Paraná, and Midwest of Rio Grande do Sul, and negative correlation in southwest of Parana state, northwest and few municipalities of southeast of Santa Catarina, and northeast and a few municipalities of central-north of Rio Grande do Sul (Fig 4A). For the same group, a small and dispersed positive spatial correlation between educational level (scholarity) and CRC mortality was present in a few municipalities in the northwest, midwest and south of Paraná and north, central-east and east of Santa Catarina. In addition, a negative correlation between scholarly and CRC mortality was present in the west region of Santa Catarina (Fig 4B).

**Fig 5:**
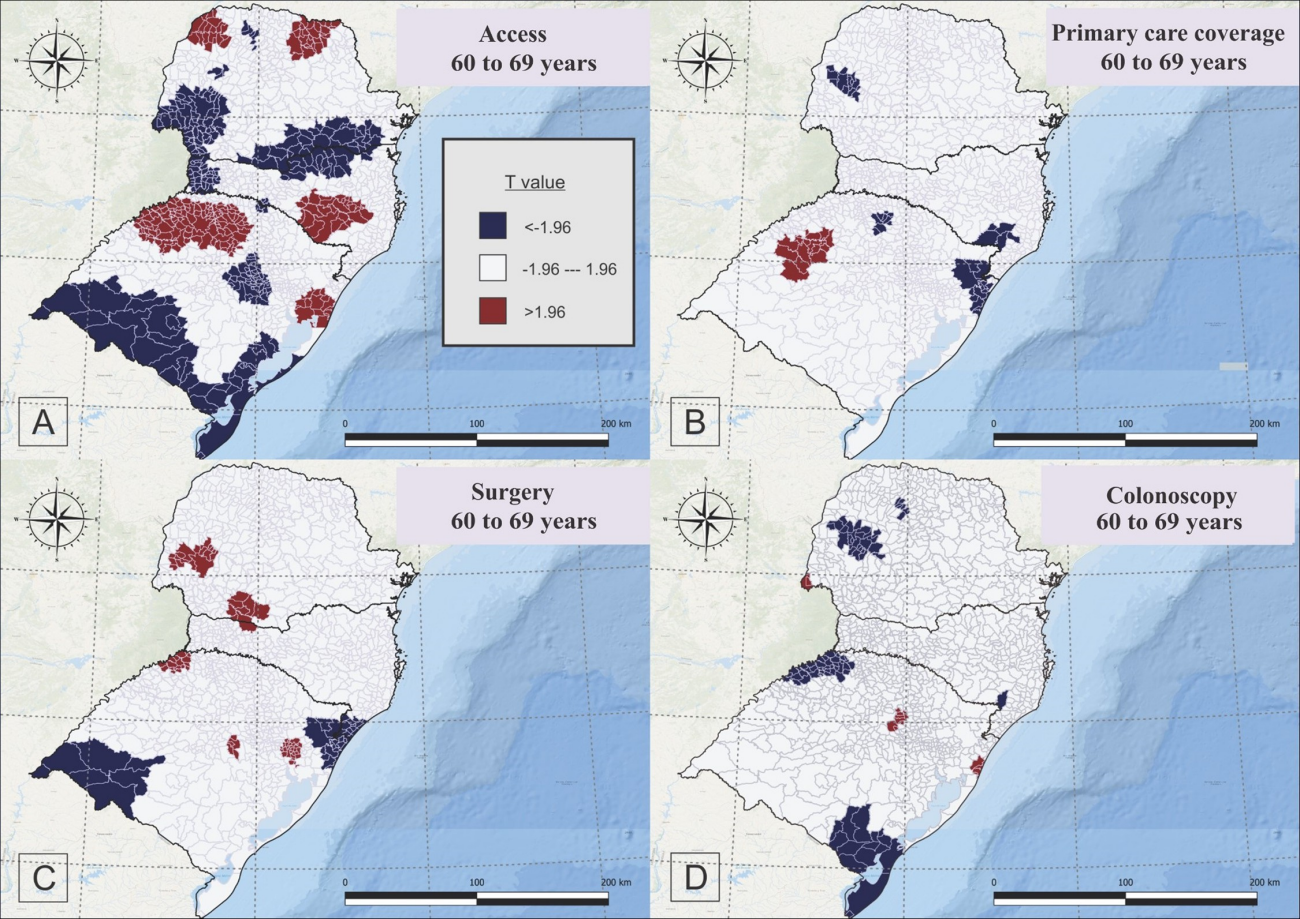
Geographically Weighted Regression for Accessibility to Oncology Center (A) primary care coverage (B), surgeries rates (C), and colonoscopy rates (D) for people aged 60 to 69 years old, who died due to colorectal cancer, in South Brazil, between 2015 and 2019.

The GWR analysis for people aged 60 to 69 years old showed a positive association with accessibility to oncology centers in the northwest and northeast of Paraná, central-east to southeast of Santa Catarina, and northwest to north and a few municipalities in the east of Rio Grande do Sul, as well as a negative correlation with CRC mortality in few municipalities in the north, west and east, southwest and south to southeast of Paraná, west and north to northeast of Santa Catarina, and central-east, few municipalities in the north, and a large portion of southwest to southeast of Rio Grande do Sul (Fig 5A).

For the same age group, the primary healthcare coverage (FHS) showed a positive correlation to CRC mortality in the west of Rio Grande do Sul, and negative correlation in a specific part of east and west of Parana, southeast of Santa Catarina and a specific point of north and northeast of Rio Grande do Sul (Fig 5B).

Surgery rates were positively related to CRC mortality in people aged 60 to 69 years in a specific region of west and south of Parana, a specific point in north of Santa Catarina, and specific points of northwest, center and northeast of Rio Grande do Sul, and negatively correlated in a specific point of east of Parana, a specific point of southeast of Santa Catarina, and Northeast and Southwest of Rio Grande do Sul (Fig 5C).

Colonoscopy rate showed a positive correlation with CRC mortality in specific points in the west of Parana, central-north and northeast of Rio Grande do Sul, and a negative correlation in specific points of north, east and west of Parana, a specific point in the southeast of Santa Catarina, a portion of northwest and south of Rio Grande do Sul (Fig 5D).

**Fig 6:**
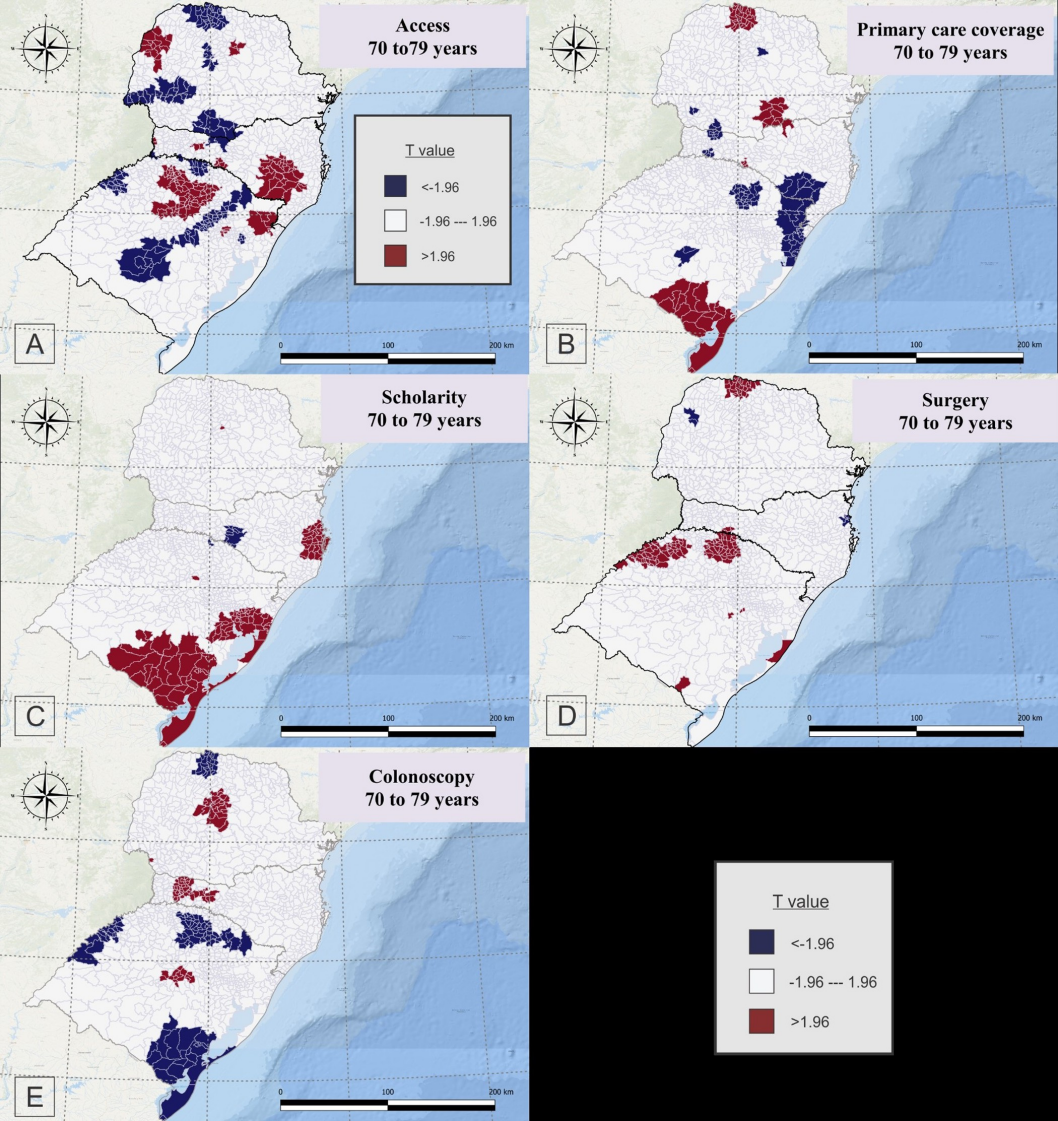
Geographically Weighted Regression for Accessibility to Oncology Center (A) primary care coverage (B), Schooling (C), Surgeries rates (D) and Colonoscopy (E) for people aged 70 to 79 years old, who died due colorectal cancer, in South Brazil, between 2015 and 2019.

For people aged between 70 to 79 years, accessibility to oncology centers showed a positive correlation with CRC mortality in the west and a specific point of northeast of Parana, few municipalities in the west, center and south regions and a large portion of southeast to center-east of Santa Catarina, and center-north and northeast of Rio Grande do Sul, and negative association in in the north, central-north, west to center, and south of Parana, a few portion of north and south of Santa Catarina, and few portion of northwest and northeast, west and east and northeast to south of Rio Grande do Sul (Fig 6A).

For the same age group, primary healthcare coverage showed a positive correlation to CRC mortality in the north and south of Parana, portions of north and south of Santa Catarina and south of Rio Grande do Sul, as well as a negative correlation in specific points of central-north and southwest of Parana, southeast and specific points of northwest and southwest of Santa Catarina, and northeast, north and specific points of central-south of Rio Grande do Sul (Fig 6B).

Educational level (schooling) had a positive correlation with CRC mortality in small point the center of Parana, in the east of Santa Catarina and East to South of Rio Grande do Sul, and negative correlation in specific points of south of Santa Catarina and a specific point in the north of Rio Grande do Sul (Fig 6C).

Surgery rates were positively related to CRC mortality in people between 70 to 79 years in specific point of north of Parana, north to west, and some points in the center, south and east of Rio Grande do Sul, and negatively related in the specific points in the west and east of Parana, specific points in the northeast to east of Santa Catarina and a specific point in the northeast of Rio Grande do Sul (Fig 6D).

Colonoscopy rates were positively related to CRC mortality in the center of Parana, west to center of Santa Catarina, and a specific point in the center of Rio Grande do Sul, and negatively related in the north of Parana, and north, west and south of Rio Grande do Sul (Fig 6E).

**Fig 7:**
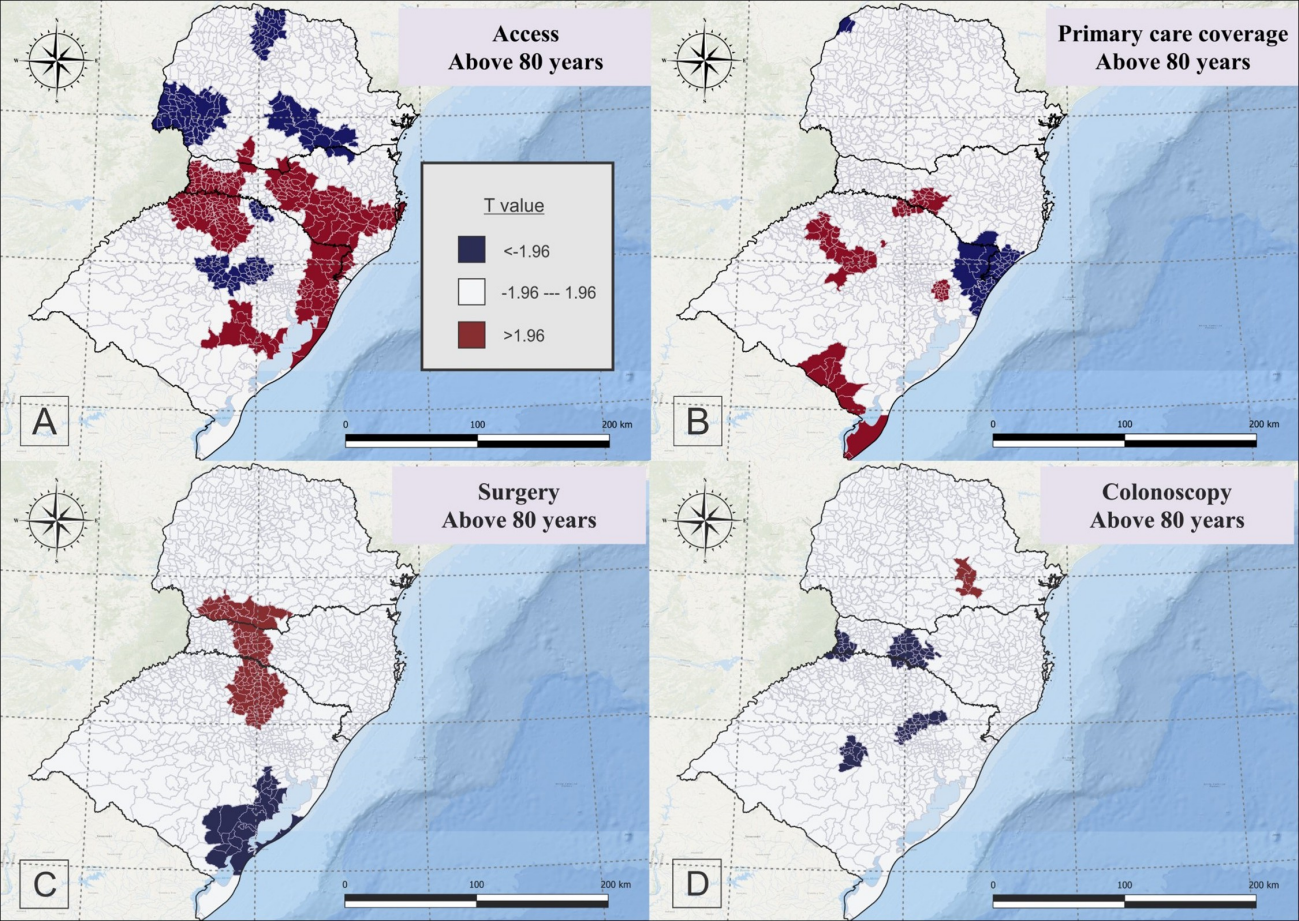
Geographically weighted regression for accessibility to oncology center (A), primary care coverage (B), surgery rates (C), and colonoscopy rates (D) for people aged 80 years old or more, who died due to colorectal cancer, in south Brazil, between 2015 and 2019.

For people aged 80+ years, the accessibility to oncology centers had a positive correlation with CRC mortality in a specific point of south of Parana, west, southwest, north, center and southeast and east of Santa Catarina, and northwest, northeast to east and center-east of Rio Grande do Sul, and negatively related in the north, southwest and central to southeast of Parana, and a specific point in the north and center of Rio Grande do Sul (Fig 7A).

For the same age group, the primary care coverage showed positive correlation with CRC mortality in a specific region of south of Santa Catarina, a specific region of north and northeast and west to center, and south of Rio Grande do Sul, and was negatively related in a specific region of northwest of Parana, southeast of Santa Catarina and northeast of Rio Grande do Sul (Fig 7B).

Surgery rates were positively related in the southwest to south of Parana, northwest to south of Santa Catarina, north of Rio Grande do Sul, and negatively related in the southeast and south of Rio Grande do Sul (Fig 7C). Colonoscopy rates were positively related to CRC mortality only in a specific part of southeast of Parana, and negatively related in specific points of west and midwest of Santa Catarina, and specific points of north, northwest, center and center-northeast of Rio Grande do Sul (Fig 7D).

## Discussion

A previous study from 2022 indicated that CRC lethality was more than double in individuals over 70 years old compared to those aged 20 to 49 years old [17], but to our knowledge, this is the first study to analyze the spatial distribution of CRC mortality in the south region of Brazil and its associations with accessibility to oncology centers and socioeconomic factors. Our results show that there was high CRC mortality in the three states of Southern Brazil over 10 years, with the highest mortality cluster in Rio Grande do Sul. In addition, the GWR regression analysis indicated that the analyzed variables related differently with CRC mortality rates according to analyzed age-groups and also spatially. However, some patterns were identified, such as the predominant negative relation of CRC mortality rate and colonoscopy rates and also with primary health coverage, and positive relation of CRC mortality rate and accessibility to oncology centers, educational level and surgery rate in some age-groups and regions.

Different studies indicated that dietary risks and tobacco consumption were some risk factors associated with CRC [50, 51]. In this context, the Rio Grande do Sul population are the greatest meat consumers in Brazil [52], and south of Brazil accounts for almost 96% of tobacco production in the country [51].

Other factors like aging, inflammatory diseases, genetics, physical inactivity and/or lack of ingestion of fruits, vegetables, and fibers can also predispose individuals to CRC, and strategies to strengthen prevention and early diagnosis can be key to decreasing CRC mortality [4]. However, reducing the CRC mortality rates is still a challenge due to the unequal distribution of healthcare services across the territory [2–24].

Strategies such as primary care programs are extremely important in disease prevention, and in the case of CRC, can influence participation in screening programs and consequently the early diagnosis and treatment of the patient [53, 54]. Screening, mainly by colonoscopy, has been effective to reduce as high as 68% of CRC deaths in Brazil [5, 7]. However, the adoption of prevention methods, diagnostic tests, and healthcare services are not evenly distributed across Brazil, creating regions with a high incidence of deaths by CRC as demonstrated in the 70 to 79 and 80+ age groups.

Our results further demonstrate that accessibility to oncology centers, educational level, and surgery rate were risk factors for CRC death in south Brazil, despite previous studies showed that lower screening, diagnostic rates, and accessibility to healthcare services in the country are centered in rural areas and among working adults with limited education, low income, or no health insurance [22, 55]. This discrepancy indicates that other factors such as the distance and difficulty of accessing specialized centers, lack of tests and screening for CRC, and lack of patient awareness are also likely to increase the number of deaths by CRC [56, 57].

Bretthauer et al. conducted a randomized trial with a total of 84,585 participants and observed that the risk of colorectal cancer at 10 years was lower among participants who were invited to undergo screening colonoscopy than among those who were not assigned to screening [58]. A recent systematic review described that adherence to CRC screening depends on the awareness of CRC diagnostic and primary care recommendations (e.g., performing colonoscopy screening) among health services [59, 60]. Moreover, guidelines from the United States recommend regular screening for CRC from 45 to 75 years old, optional screening from 76 to 85 years old, and no screening in people over 85 years old [61].

Our results showed education level was a risk factor to mortality in the age group from 50 to 79 years, with no significance in people over 80 years. Our finding disagrees with other authors [22, 55], but might be due to the fact that the analyzed education level was up to 8^th^ grade, which itself could be classified as a low education level in other studies.

An unanticipated finding of our study was the positive association between surgery rate and CRC mortality in age groups older than 60 years. We could not attribute a specific cause, though previous studies have demonstrated that CRC survival also depends on factors that could be influenced by the expertise of the surgeon and treating hospitals, such as tumor biology and staging, quality of surgical resection, lymph node involvement, and appropriate use of neoadjuvant and adjuvant therapy [60]. Overall, our results suggest that the applied health policies should aim to improve screening rates and access to health services and take into account differences across regions of South Brazil [62].

A study presented by Berg et al. [63] and Valadão et al. [64] regarded the CRC stage of the patients admitted to hospitals in Rio Grande do Sul and Rio de Janeiro, respectively. Both described that more than 60% of the population presented advanced colorectal cancer with stage III or IV at the time of diagnosis. It is public knowledge that the waiting time for diagnostic exams in the Unified Public System in Brazil can be very long. In Porto Alegre municipality, the monthly number of consultations for Proctology–Oncology ranged from 5 to 29 visits, with a waiting time of 18–34 days, and only after diagnosis of malignant neoplasm, patients can be referred to the Oncology service [63]. The significant number of patients awaiting consultation in the primary care services may indirectly reinforce the need to extend the colonoscopy exam to Unified Public System patients.

The knowledge presented in this study regarding the spatial analysis of mortality by CRC and associated factors can help governments and/or other institutions to guide public policies with minimal spending and optimal resource allocation. Further, the use of free and publicly available secondary data in this study increases transparency. In the same vein, however, potential limitations include the use of secondary data. Though DATASUS and IBGE are high-quality sources, there could be under- or over-estimations of CRC deaths and socioeconomic variables used in this study. Additional limitations are that our results are based on association analyses and cannot be used to draw causal conclusions or to generalize to the rest of Brazil or other countries. Spatial data analysis, however, is a powerful public health tool and can be applied to other contexts and to the study of other diseases worldwide.

## Conclusion

Across all age groups, a large cluster of high CRC mortality formed in the Rio Grande do Sul state of Brazil. Further, although specific factors associated with CRC mortality varied across age groups and regions, our results suggested that the presence of family health strategy teams and higher rates of colonoscopies are protective factors against colorectal cancer mortality in southern Brazil.
